# Aluminum stress signaling, response, and adaptive mechanisms in plants

**DOI:** 10.1080/15592324.2022.2057060

**Published:** 2022-04-25

**Authors:** Huabin Liu, Rong Zhu, Kai Shu, Weixiang Lv, Song Wang, Chengliang Wang

**Affiliations:** a College of Life and Health Sciences, Anhui Science and Technology University, Fengyang, China; b School of Ecology and Environment, Northwestern Polytechnical University, Xi’an, China; c Key Laboratory of Southwest China Wildlife Resources Conservation, China West Normal University, Nanchong, China; d Anhui Provincial Key Lab. of the Conservation and Exploitation of Biological Resources, School of Life Sciences, Anhui Normal University, Wuhu, China

**Keywords:** Al stress, Al tolerance, molecular regulation mechanism, phytohormones, plant growth and development

## Abstract

Over 40% of arable land in the world is acidic. Al stress has become a global agricultural problem affecting plant growth and limiting crop production in acidic soils. Plants have evolved different regulatory mechanisms of adaptation to exogenous environmental challenges, such as Al stress, by altering their growth patterns. In the past decades, several key genes involved in plant response to Al stress and the mechanism of Al detoxification have been revealed. However, the signaling pathways of plant response to Al stress and the regulatory mechanism of plant Al tolerance remain poorly understood. In this review, we summarized the findings of recent studies on the plant Al tolerance mechanism and the molecular regulation mechanism of phytohormones in response to Al stress. This review improves our understanding of the regulatory mechanisms of plants in response to Al stress and provides a reference for the breeding of Al-tolerant crops.

## 1. Introduction

As the most abundant metal element on earth, Al is widespread in the environment. It is an amphoteric metal with active chemical properties; apart from reacting with strong bases, it also dissolves in acidic solutions. In the soil, Al normally exists in the form of insoluble oxides or aluminosilicates, and in this chemical form, it exerts no toxic effects on plants.^1^ When the soil pH is below 5.5, Al and aluminide become increasingly soluble and form Al^3+^, Al(OH)^2+^, or Al(OH)_2_^+^.^2–4^ Solubilized Al, especially Al^3+^, is highly toxic to the root apex and severely restricts the ability of the root system to absorb water and nutrients in acidic soils.^5,6^ Although Al is not considered a nutrient element, previous studies have shown that low concentrations of Al^3+^ can promote plant growth.^7,8^ When the concentration of Al^3+^ in the soil reaches micromolar levels, plant growth and development are negatively affected and show symptoms of Al toxicity.^9,10^

Currently, approximately 40% of arable land in the world is acidic.^11^ With the increase in acid gases emitted by different industries as well as the increase in acid rain pollution, the threat of Al stress to plant growth and human food security is further exacerbated. Consequently, Al stress has emerged as crucial environmental issue for plants, second only to drought stress.^11^ Al causes toxicity and irreversible damage to the growth of plants, consequently affecting crop yield and quality.^12,13^ Therefore, exploring the molecular mechanisms of Al tolerance in plants is essential to improving agricultural practices, as there will be a continual demand for plants that can cope with environmental changes, as well as for increasing the production and supply of safe food.

## 2. Mechanisms of Al toxicity and adaptive response in plants

### 2.1 Al toxicity in plants at the cellular level

Roots are the main plant parts exposed to Al stress, and primary root elongation and vitality are severely inhibited by Al^3+^.^14–18^ When plants are exposed to Al stress, the most obvious symptom is the inhibition of root elongation, following which the acquisition of water and nutrients becomes limited.^8,19,20^ Consequently, plants fail to obtain sufficient nutrients and begain to manifest symptoms of nutritional deficiencies.^5,14^ Moreover, Al stress can cause programmed cell death and leaf yellowing, which leads to early plant senescence.^21,22^ Therefore, Al stress is critical factor affecting plant growth and limiting crop yield in areas with acidic soil.^23^

As mentioned above, the toxic effect of Al on plants mainly occurs through the inhibition of root elongation, which further affects plant growth and development.^24,25^ For this reason, researchers usually use roots as experimental materials to explore the response mechanisms of plants to Al stress.^25,26^ Previous studies have shown that Al^3+^ mainly affects root growth by inhibiting cell elongation and division.^17,27,28^ The root tip is considered the primary target site for Al^3+^, and the transition zone between the root tip meristem and the elongation zone is the root area most sensitive to Al stress.^8,29–31^ Therefore, the root tip region should be the focus of research on Al stress resistance mechanisms.

The toxicity of Al to plants is primarily caused by its influence on cell structure and cell life activities, especially cell wall structure and cell division.^17,27,28,32^ The cell wall serves as the first natural barrier for plants to resist harmful environments, and is vital for plant defense.^33^ It is rich in carboxyl and phosphate groups which carry a substantial amount of negative charge.^34^ Al^3+^ binds to the cell wall through cation exchange to prevent itself from binding to the plasma membrane or entering the symplasm.^34,35^ The amount of Al^3+^ binding to the cell wall is directly correlated with the damage to the plant.^33,36,37^ Studies have shown that Al^3+^ thickens the cell wall and changes its composition, hindering cell division and elongation, consequently inhibiting root elongation.^17,28,38,39^

Reactive oxygen species (ROS) are important signaling molecules in plant stress response.^40,41^ Oxidative stress is an integral aspect of the toxic effects of Al on plants.^30,42,43^ Al exposure causes the increase in ROS and leads to lipid peroxidation, resulting in cell organelles dysfunction and damage.^30,42–46^ The over-accumulation of ROS is induced by Al stress, which leads to peroxidative damage to the plasma membrane and destroys cell membrane integrity.^34,47–50^ Further studies showed that ROS accumulation and related cell dysfunction are also involved in Al-induced inhibition of cell elongation and division.^47^ Impairment of cellular function and DNA damage are major factors responsible for inhibiting root elongation.^12,51–53^ Previous research showed that the scavenging of ROS contributes to plant Al.^43^ Therefore, strategies to improve the scavenging ability of ROS and reduce the production of ROS will be a lucrative research direction to enhance plant tolerance to Al stress.

Previous studies have shown that Al also affects cell-membranes function and numerous physiological processes.^19,27,54^ Al^3+^ is a blocker of various cation channels on the cell membrane. It therefore affects the absorption of mineral elements by changing plasma membrane fluidity and structure, further interfering with the normal physiological process of plant cells.^19,27,55^ Al^3+^ competitively binds to Ca^2+^ receptors on the plasma membrane, inhibits Ca^2+^ transmembrane transport, and disrupts cytosolic Ca^2+^ homeostasis.^54,56^ In addition, Al^3+^ inhibits the absorption of K^+^ by the root system and reduces the plant potassium content, causing symptoms of K^+^ deficiency.^57^ Active transport of numerous ions is driven by H^+^ gradient established by proton pumps, such as vacuolar H^+^-pyrophosphatase (V-PPase), vacuolar H^+^-ATPase (V-ATPase), and plasma membrane (PM) H^+^-translocating adenosine triphosphatase (PM H^+^-ATPase).^18,58,59^ Al toxicity not only destroys the structure and physiological activities of plant cells, but also affects the metabolism and life processes of cells. However, further studies are required to reveal the mechanisms of Al toxicity.

### 2.2 Adaptive mechanisms of plant tolerance to Al toxicity

During their long-term evolution, plants have developed a variety of adaptation strategies to cope with Al toxicity, among which internal tolerance and external exclusion are widely considered to be the main strategies.^5,14,18^ The exclusion mechanism includes secreting organic acids (OAs) or phosphoric acid into the apoplastic space to chelate external Al.^13,18,60^ In addition to the chelation, the cell wall is considered another natural barrier for Al.^61,62^ On the other hand, the internal tolerance mechanisms involve the chelation of Al^3+^ by OAs in the cytosol, its transport, and the storage of its complexes into vacuoles.^13,18^

The cell wall is the plant’s first barrier against harmful external environments. Studies have shown that most of the Al^3+^ absorbed by plants is distributed in the cell wall.^63–65^ Cell wall polysaccharides, especially pectin, carry numerous carboxyl groups and demonstrate a strong affinity for Al^3+^.^5,27,66^ Extracellular Al^3+^ ions can bind directly to the cell wall.^61^ Xyloglucan is an important structural component of cell walls. *XTHs*, encoded xyloglucan endotransglucosylase-hydrolase, are involved in cell wall extension.^61^ *XTH31* modulates Al binding capacity by regulating the content of xyloglucan in the cell wall, thereby affecting plant sensitivity to Al toxicity.^61^ The fixation of Al on cell wall results in the sensitivity of root growth to Al toxicity.^12^ The components and structure of the cell wall are altered by Al toxicity. Excessive Al binding to the cell wall leads to the disruption of cell wall extension, thereby inhibiting cell and root elongation.^61^ Multiple genes related to cell wall synthesis or modification are involved in plant response to Al toxicity.^61,67,68^ WAK1 (cell wall-associated receptor kinase 1) co-localizes with pectin, which is critical for Al binding^69^ GRP3, a glycine-rich protein (GRP), is involved in plant response to Al stress by interacting with AtWAK1.^70^ *WAK1* overexpression or a mutation in *GPR3* results in enhanced plant Al tolerance.^69,70^ However, the molecular mechanism underlying cell wall involvement in regulating plant tolerance to Al stress remains to be elucidated.

Furthermore, studies have shown that pectin methylation in the cell wall is related to the ability of plants to resist Al^3+^ absorption.^28,33,71^ *PME* is a gene encoding pectin methylesterase in plants, and its expression level in Al-tolerant plants is significantly lower than that in Al-sensitive plants.^71^ In maize, exogenous application of pectin methylesterase resulted in the accumulation of Al^3+^ in the roots and inhibited root growth.^28,72^ The higher the methylation level, the lower the cation exchange capacity in the cell wall. This, in turn, reduces the amount of Al^3+^ bound to pectin, resulting in a decrease in the damage caused by Al stress.^28,71,72^ In addition, Al^3+^ destroys the plasma membrane structure and transmembrane ion channels by binding to plasma membrane phospholipids. This inhibits the transmembrane transport of certain ions and interferes with the ion balance in the cell, consequently affecting intracellular physiological functions.^27^

In plant roots, Al toxicity induces the secretion of chelating agents such as OAs and phosphoric acid.^18,73^ These substances chelate with Al^3+^ around the plant roots to form macromolecule chelates, thereby hampering the entry of Al^3+^ into cells and ameliorating the toxic effects of Al on plants.^18^ Plants mainly transport OAs such as malic acid and citric acid, to the cell exterior through a transport carrier on the plasma membrane to chelate Al^3+^ around the rhizosphere.^73^ The transmembrane transport of OAs is driven by the proton pump on the plasma membrane (PM H^+^-ATPase).^18,74,75^ The activity of the proton pump is significantly enhanced under Al stress, which promotes the efflux of malic and citric acids.^18^ In plants, *MATE* encodes a citric acid transporter, and *ALMT* encodes a malic acid transporter.^73,76–79^ Al stress significantly increases the expression of MATE and ALMT, promotes the secretion of citric and malic acids, and chelates Al^3+^ in the rhizosphere, thereby reducing Al toxicity stress.^73,80–84^ *TaALMT1*, the first Al-tolerance gene, was identified from wheat.^85^ Although TaALMT1 is functionally active without Al stress, its transport capability can be improved further by Al.^85–87^ In addition, *ALMT1* from other species has also been shown to improve Al tolerance in plants.^75,88,89^ Different from ALMT, MATE exhibits two distinct physiological functions in plants. Certain members of the MATE family facilitate Fe translocation while others are involved in external Al detoxification.^73,90–92^ The vacuole is the principal storage site for OAs. Malic and citric acids synthesized in the cytoplasm are transported to the vacuole, which enhances the plant’s internal tolerance to Al toxicity.^18^ In addition, plants express the ABC transporter family gene *ALS3* to promote the transport of Al^3+^ in the roots and reduce the distribution of Al^3+^ in Al-sensitive cells or tissues, thereby mitigating the effects of Al toxicity on plants.^93^

**Figure 1.**
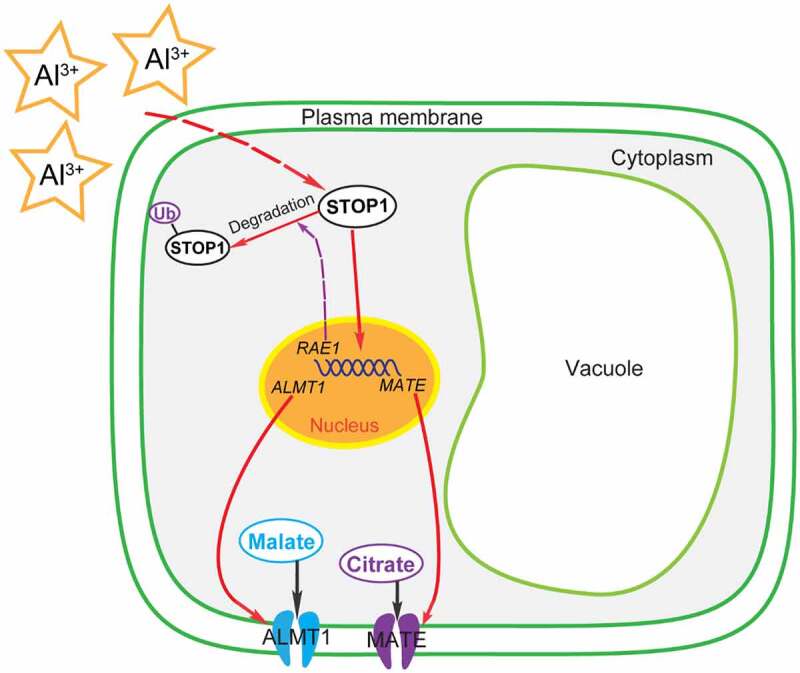
Proposed model for the regulation of malate and citrate secretion by STOP1 in response to Al stress and the proposed signaling pathway of Al-activated root malate and citrate exudation based on recent research on *Arabidopsis*.^6,18,73,94,95^ In response to Al stress, Al^3+^ signals can be perceived by the plant and trigger the accumulation of STOP1 in the cell. As a transcription factor, STOP1 upregulates the expression of *RAE1, ALMT1*, and *MATE. RAE1* reduces the amount of STOP1 by promoting the ubiquitination (Ub) and degradation of STOP1.^6,18,95^ Al-activated excretion of malate and citrate occurs through the PM localized transporters of ALMT1 and MATE, respectively. The secretion of OAs plays a critical role in plant Al tolerance through the chelation of external Al.

Previous studies showed that STOP1, a C2H2-type transcription factor, plays an important role in plant Al resistance;^80,86^ it enhances plant tolerance to Al by regulating the expression of Al-resistance genes, including *ALS3, MATE*, and *ALMT1* (Figure 1).^6,73,80,86^ Al stress promotes the accumulation of STOP1 in cells.^6^ STOP1 directly acts on the promoter region of *RAE1* to augment the expression of *RAE1*, and RAE1 interacts with the STOP1 protein through the ubiquitinated 26S protease pathway to promote the degradation of STOP1.^6^ Therefore, a feedback regulation loop is formed between RAE1 and STOP1 (Figure 1).^6^

The secretion of malate and citrate by root cells plays a crucial role in plant Al tolerance and contribute to its detoxification.^73^ Al-activated malate and citrate exudation were found to be affected by the loss of the STOP1 function, and the *stop1* mutant showed increased sensitivity to Al.^73,80^ To cope with Al toxicity, plants secrete OAs to chelate external Al and/or regulate the expression of related genes responsible for plant Al-resistance (Table 1).^6,18,25,73,77,78,85,90,93–105^ Although plants have different regulatory mechanisms and adaptive strategies to cope with Al toxicity, the molecular mechanisms underlying these strategies remain largely unclear.

**Table 1.** Related genes responsible for Al-activated secretion of OAs and plant Al-resistance

Gene	GenBank accession	Gene type	The subcellular location	Species	Gene expression patterns	References
*AtMATE*	*At1g51340*	Citrate transporter	Plasma membrane	*Arabidopsis thaliana*	Al-induced up-regulation	^6,73^
*ZmMATE1*	*Zm00001d035115*	Citrate transporter		*Zea mays* (Maize)	Al-induced up-regulation	^25,78^
*SbMATE*	*EF611342*	Citrate transporter	Plasma membrane	*Sorghum bicolor* (Sorghum)	Al-induced up-regulation	^96^
*HvAACT1/HvMATE1*	*AB331641*	Citrate transporter	Plasma membrane	*Hordeum vulgare* (Barley)		^97^
*TaMATE1*	*BE605049*	Citrate transporter, belonging to the multidrug and toxin efflux (MATE) gene family		*Triticum aestivum* (Wheat)		^98^
*FeMATE1*	*comp57549_c0*	Multi-drug and toxic compound extrusion	Plasma membrane	*Fagopyrum esculentum* (*Buckwheat*)	Al-induced up-regulation	^99,100^
*FeMATE2*	*comp55339_c0*	Multi-drug and toxic compound extrusion	Trans-Golgi and Golgi	*Fagopyrum esculentum* (*Buckwheat*)	Al-induced up-regulation and leaves	^99,100^
*OsFRDL4*	*Os01g0919100*	Citrate transporter, belongs to the multidrug and toxic compound extrusion (MATE) family	Plasma membrane	*Oryza sativa* (Rice)	Al-induced up-regulation	^90^
*AtALMT1*	*At1g08430*	Al-activated malate transporter 1	Plasma membrane	*Arabidopsis thaliana*	Al-induced up-regulation	^18,73,77^
*TaALMT1*	*AB081803*	Al-activated efflux of malate	Plasma membrane	*Triticum aestivum* (Wheat)		^85^
*VHA-a2*	*At2g21410*	The vacuolar H1-ATPase, vacuolar H1-translocating adenosine triphosphatase (H1-ATPase) subunit a2		*Arabidopsis thaliana*	Al-induced down- regulation	^18^
*VHA-a3*	*At4g39080*	The vacuolar H1-ATPase, vacuolar H1-translocating adenosine triphosphatase (H1-ATPase) subunit a3		*Arabidopsis thaliana*	Al-induced down- regulation	^18^
*ESD4*	*At4g15880*	The small ubiquitin-like modifier (SUMO) protease	Nuclear rim	*Arabidopsis thaliana*		^94,101,102^
*HPR1*	*At5g09860*	Hyperrecombination protein 1 (HPR1), a subunit of the THO/TREX complex	Nucleus	*Arabidopsis thaliana*		^95^
*RAE1*	*At5g01720*	The F-box protein Regulation of AtALMT1 Expression 1 (RAE1)	Nucleus	*Arabidopsis thaliana*	Al-induced up-regulation	^6,103^
*RAH1*	*At5g27920*	RAE1 homolog 1	Nucleus	*Arabidopsis thaliana*	Al-induced up-regulation	^103^
*STOP1*	*At1g34370*	The C2H2-type zinc finger transcription factor sensitive to proton rhizotoxicity 1		*Arabidopsis thaliana*	*STOP1* transcription is not affected by Al stress, Al stress triggers STOP1 protein accumulation.	^6^
*STAR1*	*AB253626*	a bacterial-type ATP binding cassette (ABC) transporter, sensitive to Al rhizotoxicity1	The vesicle membrane	*Oryza sativa* (Rice)	Al-induced up-regulation	^104^
*STAR2*	*AB379845*	a bacterial-type ATP binding cassette (ABC) transporter	The vesicle membrane	*Oryza sativa* (Rice)	Al-induced up-regulation	^104^
*ALS1*	*At5g39040*	The ATP-binding cassette (ABC) transporter, aluminum-sensitive 1	Vacuolar membrane	*Arabidopsis thaliana*		^105^
*ALS3*	*At2g37330*	The ATP-binding cassette (ABC) transporter-like protein	Plasma membrane	*Arabidopsis thaliana*		^6,93^

Previous studies have shown that the ability of plants to tolerate Al stress is related to rhizosphere pH.^3^ Al solubility increases under acidic conditions, whereas it decreases significantly in a weakly alkaline environment. In addition to the chelation of Al^3+^ by OAs secretion, the solubility of Al can also be decreased by maintaining a higher pH of the rhizosphere and reducing the entry of Al^3+^ into the plant. This strategy is considered an effective method to enhance plant Al resistance. Therefore, a higher pH environment is beneficial for enhancing plant Al tolerance and alleviating its toxicity.^106,107^

### 2.3 Hormone signaling in plant Al stress response: ethylene and auxin as the key factors

Phytohormones play key roles in plant growth regulation in response to Al stress (Table 2).^8,19,20,25,108–112^ Al stress upregulates *TAA1* and *YUCs* (*YUC3/5/7/8/9*) in the roots, which promotes a localized increase in auxin synthesis and causes root growth inhibition.^8,20^ Ethylene has been reported to regulate plant Al tolerance through crosstalk with auxin signaling.^19,20^ Al exposure upregulates the expression of ethylene biosynthesis-related genes, such as *ACSs* and *ACOs*, thereby promoting ethylene synthesis.^19^ As a signaling molecule, ethylene activates specific expression of the transcription factors *EIN3* and *EIL1* in the root apex transition zone.^20^ EIN3 directly binds to the promoters of *YUC9* and activates its expression.^20^ The expression of *PIF4* is also regulated by EIN3 and EIL1; moreover, the bHLH transcription factor *PIF4* affects auxin biosynthesis and signaling by directly regulating the expression of *YUC5, YUC8*, and *YUC9*.^20^ In addition, ethylene also upregulates the expression of *TAA1* and promotes the local biosynthesis of auxin in the root apex transition zone to enhance the inhibition of root growth.^8^ Therefore, the accumulation of auxin induced by Al stress is regulated by ethylene signaling.^19^

**Table 2.** Hormone signaling-related genes in plant response to Al stress

Gene	GenBank accession	Gene type	The subcellular location	Species	Gene expression patterns	References
*ACS2*	*AT1G01480*	Ethylene synthesis genes		*Arabidopsis thaliana*	Al-induced up-regulation	^19^
*ACS4*	*AT2G22810*	Ethylene synthesis genes		*Arabidopsis thaliana*	Al-induced up-regulation	^19^
*ACS6*	*AT4G11280*	Ethylene synthesis genes		*Arabidopsis thaliana*	Al-induced up-regulation	^19^
*ACO1*	*AT2G19590*	Ethylene synthesis genes		*Arabidopsis thaliana*	Al-induced up-regulation	^19^
*ACO2*	*AT1G62380*	Ethylene synthesis genes		*Arabidopsis thaliana*	Al-induced up-regulation	^19^
*EBS*		Ethylene reporter, a synthetic EIN3-responsive promoter		*Arabidopsis thaliana*	Al-induced up-regulation	^19^
*EIL1*	*AT2G27050*	Ethylene signaling, transcription factors		*Arabidopsis thaliana*	Al-induced up-regulation	^20^
*EIN3*	*AT3G20770*	Ethylene signaling, ethylene-insensitive 3 (EIN3)		*Arabidopsis thaliana*	Al-induced up-regulation	^20^
*DR5*		Auxin-responsive marker		*Zea mays* (Maize)	Al-induced down-regulation	^25^
*DR5*		Auxin-responsive marker		*Arabidopsis thaliana*	Al-induced up-regulation	^19^
*TAA1*	*AT1G70560*	Auxin biosynthesis, Trp aminotransferase		*Arabidopsis thaliana*	Al-induced up-regulation	^8^
*YUC3*	*AT1G04610*	Auxin biosynthesis		*Arabidopsis thaliana*	Al-induced up-regulation	^20^
*YUC5*	*AT5G43890*	Auxin biosynthesis		*Arabidopsis thaliana*	Al-induced up-regulation	^20^
*YUC7*	*AT2G33230*	Auxin biosynthesis		*Arabidopsis thaliana*	Al-induced up-regulation	^20^
*YUC8*	*AT4G28720*	Auxin biosynthesis		*Arabidopsis thaliana*	Al-induced up-regulation	^20^
*YUC9*	*AT1G04180*	Auxin biosynthesis		*Arabidopsis thaliana*	Al-induced up-regulation	^20^
*PIN1*	*AT1G73590*	Auxin efflux carriers	Plasma membrane	*Arabidopsis thaliana*	Al-induced ectopically up-regulated	^108^
*PIN2*	*AT5G57090*	Auxin efflux carriers	Plasma membrane	*Arabidopsis thaliana*	Al-induced up-regulation	^19,108^
*OsPIN2*	*Os06g44970*	Auxin efflux carriers	Plasma membrane	*Oryza sativa* (Rice)	Al-induced up-regulation	^109^
*PIN3*	*AT1G70940*	Auxin efflux carriers	Plasma membrane	*Arabidopsis thaliana*	Al-induced ectopically up-regulated	^108^
*PIN4*	*AT2G01420*	Auxin efflux carriers	Plasma membrane	*Arabidopsis thaliana*	Al-induced ectopically up-regulated	^108^
*PIN7*	*AT1G23080*	Auxin efflux carriers	Plasma membrane	*Arabidopsis thaliana*	Al-induced ectopically up-regulated	^108^
*AUX1*	*AT2G38120*	Auxin influx carriers	Plasma membrane	*Arabidopsis thaliana*	Al-induced ectopically up-regulated	^19,108^
*LAX1*	*AT5G01240*	Auxin influx carriers	Plasma membrane	*Arabidopsis thaliana*	Al-induced ectopically up-regulated	^108^
*LAX2*	*AT2G21050*	Auxin influx carriers	Plasma membrane	*Arabidopsis thaliana*	Al-induced ectopically up-regulated	^108^
*ZmPGP1*	*GRMZM2G315375*	Auxin efflux carrier P-glycoprotein		*Zea mays* (Maize)	Al-induced up-regulation	^25^
*ARF7*	*AT5G20730*	Auxin response factors		*Arabidopsis thaliana*	Al-induced up-regulation	^110^
*ARF10*	*AT2G28350*	Auxin response factors (ARFs), ARF10 is important in the regulation of cell wall modification–related genes		*Arabidopsis thaliana*		^8^
*ARF16*	*AT4G30080*	auxin response factors (ARFs), ARF16 is important in the regulation of cell wall modification–related genes		*Arabidopsis thaliana*		^8^
*ZmIAA2*	*Zm00001d033976*	Auxin-responsive genes		*Zea mays* (Maize)	Al-induced down- regulation	^25^
*ZmIAA10*	*Zm00001d041416*	Auxin-responsive genes		*Zea mays* (Maize)	Al-induced down- regulation	^25^
*ZmIAA21*	*Zm00001d013302*	Auxin-responsive genes		*Zea mays* (Maize)	Al-induced down- regulation	^25^
*ZmGH3*	*Zm00001d011377*	Auxin-responsive genes		*Zea mays* (Maize)	Al-induced down- regulation	^25^
*ARR3*	*AT1G59940*	CK-induced genes		*Arabidopsis thaliana*	Al-induced up-regulation	^110^
*ARR4*	*AT1G10470*	CK-induced genes		*Arabidopsis thaliana*	Al-induced up-regulation	^110^
*TCSn*		CK signaling, Two Component Signaling Sensor new (TCSn)		*Arabidopsis thaliana*	Al-induced up-regulation	^110^
*IPT1*	*AT1G68460*	Cytokinin biosynthesis, adenosine phosphate isopentenyl-transferases		*Arabidopsis thaliana*	Al-induced up-regulation	^110^
*IPT3*	*AT3G63110*	Cytokinin biosynthesis		*Arabidopsis thaliana*	Al-induced up-regulation	^110^
*IPT5*	*AT5G19040*	Cytokinin biosynthesis		*Arabidopsis thaliana*	Al-induced up-regulation	^110^
*IPT7*	*AT3G23630*	Cytokinin biosynthesis		*Arabidopsis thaliana*	Al-induced up-regulation	^110^
*PIF4*	*AT2G43010*	The basic helix–loop–helix transcription factors, Phytochrome-interacting factor 4 (PIF4)		*Arabidopsis thaliana*	Al-induced up-regulation	^20^
*COI1*	*AT2G39940*	Jasmonate (JA) receptor, Coronatine Insensitive 1		*Arabidopsis thaliana*	Al-induced up-regulation	^111^
*MYC2*	*AT1G32640*	JA signaling regulator		*Arabidopsis thaliana*	Al-induced up-regulation	^111^
*AOS*	*AT5G42650*	JA biosynthesis related genes		*Arabidopsis thaliana*	Al-induced up-regulation	^111^
*AOC3*	*AT3G25780*	JA biosynthesis related genes, Allene Oxide Cyclase 3		*Arabidopsis thaliana*	Al-induced up-regulation	^111^
*OPR3*	*At2g06050*	JA biosynthesis related genes, Oxophytodienoate-reductase 3		*Arabidopsis thaliana*	Al-induced up-regulation	^111^

Recent studies have shown that the polar transport of auxin is also involved in plant response to Al stress.^109^ Ethylene production was found to be induced by Al^3+^, which acts as a signal to disrupt polar auxin transport by upregulating the expression of AUX1 and PIN2 auxin transporters, leading to auxin accumulation in the roots and inhibiting root growth.^19^ Overexpression of the auxin efflux carrier OsPIN2 can alleviate Al-induced damage to the roots, which is a consequence of the decrease in extracellular Al^3+^ binding to the cell walls and reduced Al-targeted peroxidative cellular damage.^109^ In maize, the auxin efflux carrier ZmPGP1 is involved in regulating auxin distribution in the root response to Al stress. *ZmPGP1* expression was induced by Al treatment, but the accumulation of auxin was reduced in root tips.^25^

Although auxin plays an important role in plant response to Al stress, its regulation mechanism varies completely among different plant species.^8,25^ In *Arabidopsis*, Al stress induces the biosynthesis and accumulation of auxin in the root apex transition zone, and excessive auxin inhibits root growth.^8^ However, in maize, Al stress reduces auxin accumulation and inhibits root growth.^25^ These two distinct actions of auxin imply that the auxin regulation mechanisms differ among plant species; however, their molecular background remains unclear.^8,20,25^

**Figure 2.**
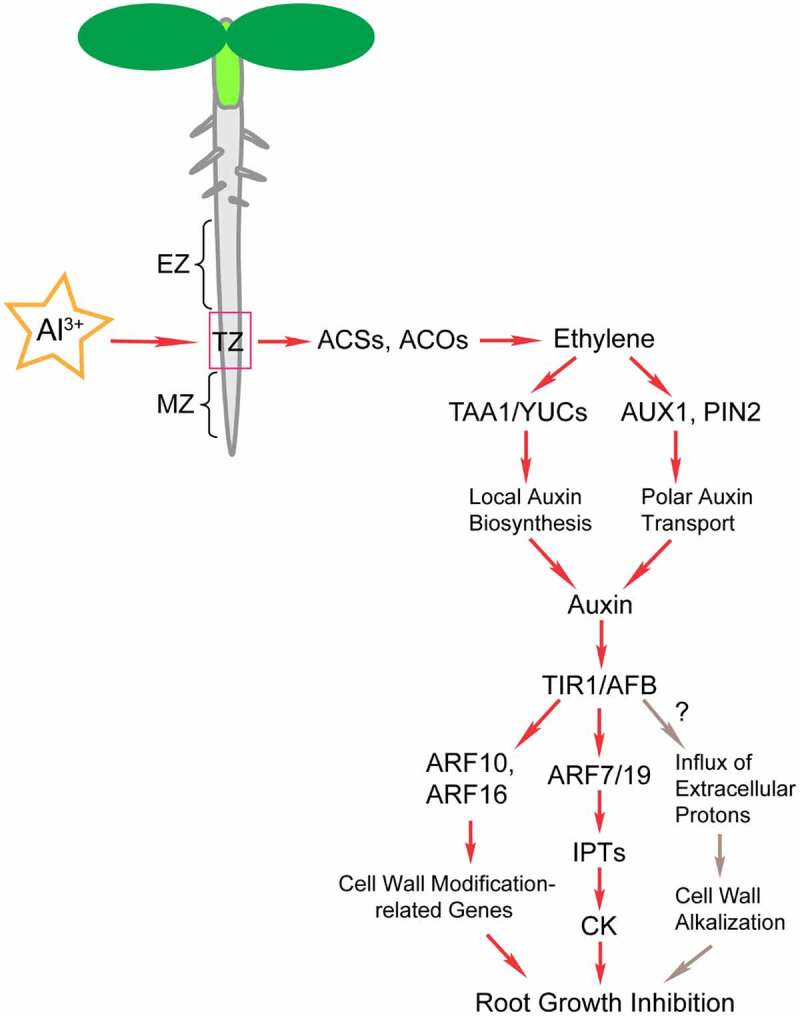
Schematic representation of ethylene- and auxin-mediated regulation of root growth inhibition in response to Al stress. The proposed hormone signaling pathway under Al stress was based on recent research on plants.^8,19,20,87,108,110,111,114,115^ The root tip is considered the main site that identifies Al toxicity. The transition zone (TZ) between the meristem and the elongation zone of the root apex is the most sensitive area for plants to perceive Al stress. Al stress induces auxin response in the root TZ, which is dependent on the ethylene signaling pathway. Al^3+^ was found to upregulate the expression of ACSs and ACOs and promote ethylene biosynthesis.^19^ Ethylene promotes local auxin accumulation through TAA1- and YUCs-mediated local auxin biosynthesis.^8,20,110^ In addition, ethylene promotes local auxin accumulation through AUX1- and PIN2-mediated polar auxin transport, resulting in root growth inhibition.^19,108^ ARF-mediated auxin signaling controls the Al-induced inhibition of root growth by regulating IPT-dependent cytokinin biosynthesis and cell wall modification-related genes.^8,110,111^

TIR1/AFB-mediated auxin signaling pathways play vital roles in regulating root elongation.^113,114^ However, there are conflicting experimental results concerning TIR1/AFB signaling in root growth under Al stress.^4,8,115^ Previous studies have shown that *tir1-1* and *tir1-1;afb2-1;afb3-1* mutants and wild type (WT) did not differ significantly in Al-induced root growth inhibition.^4^ However, Yang et al. (2014) found that the Al stress-induced auxin signals were significantly decreased by PEO-IAA (specific antagonists to block TIR1/AFB signaling) treatment.^8^ Consistently, TIR1/AFB is involved in the regulation of barley root growth inhibition under Al stress.^115^ These contradictory experimental results might be due to the different pH values of the AlCl_3_ solutions used in different experiments. The pH 7.0 was used in the former experiment^4^ while pH 4.3 and 5.0 was used in the other two experiments,^8,115^ respectively. Al^3+^ is mainly formed at pH≤5.0, whereas at pH 7.0 Al(OH)_3_ is predominant.^4^ Therefore, different forms of aluminum present lead to distinct results. Based on these results, it is inferred that Al-induced inhibition of root growth is regulated by TIR1/AFB-mediated auxin signaling pathways (Figure 2).^87,108,113,115^ Moreover, whether the known signaling pathway of TIR1/AFB-mediated apoplast alkalization is also involved in Al-induced root growth inhibition requires further study (Figure 2).

As transcription factors, auxin response factors (ARFs) are involved in auxin signaling downstream of TIR1/AFB. The auxin-regulated root growth inhibition induced by Al stress is mainly mediated by ARFs, which activates the expression of auxin response genes.^8,20,111^ ARF7 promotes cytokinin biosynthesis by upregulating the expression of *IPT5* and *IPT7*, whereas ARF10 and ARF16 are involved in Al-induced inhibition of root growth by regulating the expression of cell wall modification-related genes.^8,111^

In summary, Al-induced ethylene production is involved in auxin signaling to control root elongation under Al stress (Figure 2). Although studies have shown that exogenous application of auxin can increase the expression of *ALMT1*, malate exudation was not affected by its application.^116^ The cumulative evidence indicates that auxin and OAs exudation independently regulate the Al-induced inhibition of root growth.^8^

## 3. Conclusions

Al stress is a major constraint for plant growth and crop yield in acidic soils. Therefore, over the past decade, studies aimed at elucidating the physiological and molecular mechanisms underlying plant tolerance to Al toxicity have attracted intense research interest. To cope with Al toxicity, many plant species have evolved various mechanisms to survive in unfavorable environments. There are two adaptive mechanisms that enable plants to withstand Al stress in acidic soils: external Al exclusion and internal Al tolerance.^14,73^ The mechanism underlying internal Al tolerance involves Al fixation in the cell wall, Al chelation by OAs in the cytosol, or Al sequestration into the vacuole. The exclusion mechanism involves the secretion of OAs from plant roots for Al^3+^ chelation. Although the responses of different plant species to Al share the same or similar regulatory mechanisms, there are still slight differences among different plant species, which depend on the signaling pathway activated by Al. Further research will help reveal species-specific mechanisms of plant Al tolerance. The Al tolerance phenotypes are the result of both environmental and genetic factors. In agricultural practices, two methods are used to overcome the threat of Al toxicity and improve plant tolerance to Al stress. Furthermore, the low pH values of acidic soils can be improved by applying alkaline substances such as CaO or Ca(OH)_2_; however, this requires considerable manpower and material resources. This issue should instead be tackled by planting Al-tolerant species or by improving cultivars through molecular-assisted plant breeding.

From the perspective of coping with changes in environmental conditions, breeding Al-tolerant and Al-insensitive plant species is the most effective and economical way to improve their ability to cope with Al stress. Exploring the response mechanism of different plant species to Al stress will help us understand the different pathways of Al tolerance. Using transcriptome analysis and genetic engineering technology to identify genes related to Al stress and improve plant Al tolerance via transgenic technology will be one of the most effective methods for breeding Al-tolerant plants.

## Abbreviations

Al: Aluminum; ROS: Reactive oxygen species; V-PPase: Vacuolar H^+^-pyrophosphatase; V-ATPase: Vacuolar H^+^-ATPase; PM: Plasma membrane; H^+^-ATPase: H^+^-translocating adenosine triphosphatase; OAs: Organic acids.
